# Is amino acid racemization a useful tool for screening for ancient DNA in bone?

**DOI:** 10.1098/rspb.2009.0563

**Published:** 2009-06-03

**Authors:** Matthew J. Collins, Kirsty E. H. Penkman, Nadin Rohland, Beth Shapiro, Reimer C. Dobberstein, Stefanie Ritz-Timme, Michael Hofreiter

**Affiliations:** 1 BioArCh, Departments of Biology, Archaeology and Chemistry, University of York, York, UK; 2 Max Planck Institute for Evolutionary Anthropology, 04013 Leipzig, Germany; 3 Department of Biology, Penn State University, University Park, PA 16802-5301, USA; 4 Institute of Legal Medicine, University of Düsseldorf, Moorenstraße 5, 40225 Düsseldorf, Germany

**Keywords:** aspartic acid racemization, ancient DNA, collagen, bone, screening

## Abstract

Many rare and valuable ancient specimens now carry the scars of ancient DNA research, as questions of population genetics and phylogeography require larger sample sets. This fuels the demand for reliable techniques to screen for DNA preservation prior to destructive sampling. Only one such technique has been widely adopted: the extent of aspartic acid racemization (AAR). The kinetics of AAR are believed to be similar to the rate of DNA depurination and therefore a good measure of the likelihood of DNA survival. Moreover, AAR analysis is only minimally destructive. We report the first comprehensive test of AAR using 91 bone and teeth samples from temperate and high-latitude sites that were analysed for DNA. While the AAR range of all specimens was low (0.02–0.17), no correlation was found between the extent of AAR and DNA amplification success. Additional heating experiments and surveys of the literature indicated that d/l Asx is low in bones until almost all the collagen is lost. This is because aspartic acid is retained in the bone within the constrained environment of the collagen triple helix, where it cannot racemize for steric reasons. Only if the helix denatures to soluble gelatin can Asx racemize readily, but this soluble gelatine is readily lost in most burial environments. We conclude that Asx d/l is not a useful screening technique for ancient DNA from bone.

## 1. Introduction

Ancient DNA studies are conducted upon an ever-growing number of species (Pääbo *et al*. 2004) and, increasingly, the move is away from phylogenetic studies that require few specimens and towards population genetics and phylogeographic research, which require tens to hundreds of specimens (e.g. Hofreiter *et al*. 2004; Shapiro *et al*. 2004). Such work often results in considerable physical damage to the ancient samples.

Set in this context, a screening method that does minimal damage to the samples and offers reasonable prediction of DNA survival—or, better still, amplification success—would be very valuable. Amino acid analysis fulfils the first condition as it requires less than 10 mg of bone, and in a pioneering investigation it was shown that DNA survival could be predicted by measuring the extent of aspartic acid racemization (AAR; Poinar *et al*. 1996). Although other screening methods have been proposed, including histology (Colson *et al*. 1997; Jans *et al*. 2004), mineral alteration (Gotherstrom *et al*. 2002), mass spectrometry (Poinar & Stankiewicz 1999), collagen alteration (Koon *et al*. 2003) and osteocalcin analysis (Smith *et al*. 2005), none of these has been widely adopted.

Despite its widespread citation and relatively widespread application, the analysis of Poinar *et al*. (1996) stands alone; there has only been one re-assessment of the validity of the approach (Fernández *et al*. 2009) and this attempted the difficult challenge of validating amplification of human DNA. Here, we take advantage of two large ancient DNA datasets (Hofreiter *et al*. 2004; Shapiro *et al*. 2004) and assess the extent of racemization in animal bones in comparison with DNA amplification success. To resolve the question of the utility of d/l Asx as a screening tool for ancient DNA survival, we examined 91 bones for which both amino acid and DNA preservation were assessed via HPLC and PCR analyses, respectively. The work was conducted in three separate laboratories at the University of York (UoY), University of Düsseldorf (UoD) and Max Planck Institute for Evolutionary Anthropology (EVA), Leipzig.

## 2. Materials and methods

Modern bovine bone compacta was obtained from a local butcher (UoD). Ancient bison bones (provided by the University of Oxford) originated from high-latitude open air sites ranging in age from modern to > 60 kyr ago (Shapiro *et al*. 2004). Ancient cave hyena samples were obtained primarily from cave sites, some of which are reported in Hofreiter *et al*. (2004) and Rohland *et al*. (2005). Archaeological (human, horse and cattle) bone was obtained from a variety of archaeological sites in northwestern Europe. Full details of samples are given in the electronic supplementary material, table S1.

### (a) Heating experiments

#### (i) Preparation of modern bone samples for heating experiments (UoD)

Pieces of defleshed compact bone were washed overnight in 15 per cent NaCl containing protease inhibitors (66 g 6-amino-*n*-hexanic acid, 3.9 g benzamidine HCl, 625 mg *N*-ethylmaleimide, 522 mg phenylmethylsulfonyl fluoride dissolved in 1 l of distilled water; Takagi & Veis 1984), extracted using ethanol/ether (volumes 3 : 1) for 15 min to remove lipophilic substances and again washed in 2 per cent SDS solution containing protease inhibitors for 1 h. The samples were exhaustively rinsed in double-distilled water and then lyophilized.

#### (ii) Preparation of total bone fraction from ancient DNA studies (UoY and EVA)

Ancient bone samples were surface cleaned using tungsten carbide rotary tools before sampling. Bone was powdered and amino acid racemization analyses (of total bone) were prepared and conducted at York and Leipzig. Briefly, bone powder was dissolved in 7 M HCl at 4°C, hydrolyzed in the same solution for precisely 6 h at 110°C and then dried *in vacuo*.

#### (iii) Preparation of soluble and insoluble ‘collagen’ fractions from ancient bone samples (UoD)

The extent of racemization in both acid-soluble (gelatine) and acid-insoluble (collagen) fractions was analysed (cf. Matsu'ura & Ueta 1980). Bone powder was demineralized overnight in 1 M HCl (10 ml HCl per 500 mg bone powder) at 4°C. The soluble fraction was separated from the insoluble collagenous fraction by centrifugation, washed until neutral pH (all washings added to the soluble fraction) and then both samples were dried *in vacuo*; collagen yields were roughly estimated using the relationship between measured weight of dry insoluble fraction and dry weight of total tissue.

#### (iv) Racemization analysis

The extent of amino acid racemization was determined by using fluorescent derivatization of hydrolysates of powder, using two different HPLC-based methods in three separate laboratories: UoY, UoD and EVA.

UoY and UoD: Hydrolysates were rehydrated in 0.01 M HCl, containing 0.01 mM l-homo-arginine as an internal standard, and 0.77 mM NaN_3_ to inhibit bacterial growth. Samples were derivatized with isobutyryl-l-cysteine and *o*-phthaldialdehyde, separated using rHPLC (Hypersil BDS 5 µm, 250 × 5 mm) and detected by fluorescence (230/445 nm); for full details, see Dobberstein *et al*. (2008) and Penkman *et al*. (2008).

EVA: Hydrolysates were rehydrated in 1 ml 10 mM sodium borate (B_4_O_7_Na*_2_10H_2_O, pH 9) by agitation for one day, then derivatized using *o*-phthaldialdehyde as described in Zhao & Bada (1995) and Poinar *et al*. (1996).

## 3. Results

### (a) Reliability of amino acid d/l and concentration estimates

Previous reports presuppose consistency between d/l Asx within individual bones. Here, we test this assumption in intra-sample and intra-laboratory analyses. Comparison of the analytical precision of d/l Asx measurements from blind independent analysis of the same hydrolysate of experimentally heated bone between laboratories (UoD and UoY; *R*^2^ > 0.99, *n* = 34) was similar to that within laboratories (*R*^2^ > 0.99, *n* > 20; electronic supplementary material, fig. S1*a*). Precision on concentration estimation (which involved summing all the resolved d and l peaks) was lower, but still highly correlated (*R*^2^ = 0.99, *n* = 34; electronic supplementary material, fig. S2*a*).

In comparison with the good analytical precision achieved from the same hydrolysate, within and among our laboratories, within-bone reproducibility was poor. Two different samples of the same bone analysed in different laboratories displayed low correlation (*R*^2^ = 0.50, *n* = 19), as did subsamples of the same coarse powder subject to different hydrolysis times analysed in the same laboratory (UoY) (*R*^2^ = 0.47, *n* = 20). Concentration displayed similarly poor correlation when prepared from the same powder (*R*^2^ = 0.51, *n* = 20) and no correlation (*R*^2^ = 0.10, *n* = 20) when separate pieces from the same bone were analysed (electronic supplementary material, fig. S2*b*,*c*).

### (b) Amplification success versus d/l Asx

**Figure 1.**
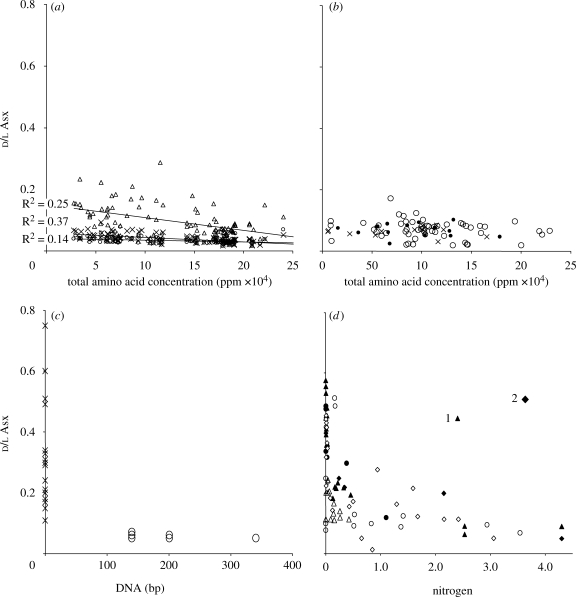
Relationship between amino acid concentration and extent of racemization. (*a*) Comparison of the d/l ratios of total bone (cross), the insoluble collagen fraction (open circle) and the soluble fraction (open triangle) against concentration (archaeological bone samples). (*b*) d/l Asx of total bone extracts plotted against concentration (aDNA sample set). Symbols refer to the ability to amplify aDNA: open circle, amplified readily; filled circle, amplified with difficulty; cross, failed to amplify. (*c*) The original Poinar *et al*. (1996) data plotted as fragment length versus d/l Asx (open circle, amplified; cross, failed to amplify). (*d*) Protein (nitrogen content) versus d/l Asx. Note the L-shaped nature of the plot, as seen also in (*c*). Data from: filled diamond, Hare (1980); filled triangle, Kessels & Dugworth (1980); open circle, Lajoie *et al*. (1980); filled circle, Prior *et al*. (1986); open diamond, Taylor *et al*. (1989); open triangle, El Mansouri *et al*. (1996). Two values that fall outside this trend are from arid environments: (1) Egyptian mummy; (2) Buhen Horse Sudan.

**Figure 2.**
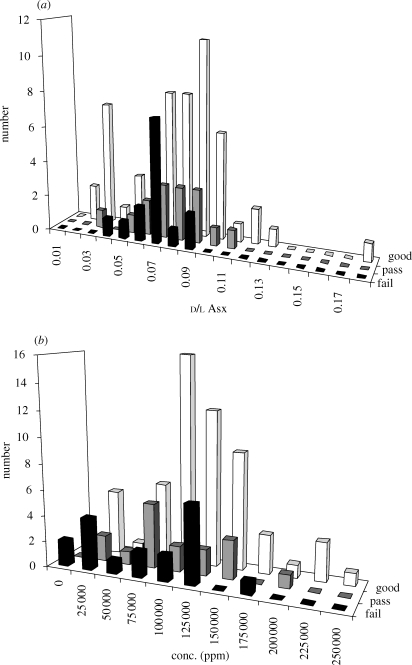
Comparison between amino acid racemization and DNA amplification. (*a*) Plot of Asx d l versus amplification success (black = fail, grey = poor, white = good). (*b*) Plot of amino acid concentration versus amplification success (black = fail, grey = poor, white = good).

d/l Asx values were obtained for 91 bones from two ancient DNA studies: Hofreiter *et al*. (2004; predominately hyena bones and teeth from cave sites) and Shapiro *et al*. (2004; bison bones from permafrost) and compared with respect to PCR success. Of these, DNA was amplified from 57 with little difficulty (table 1; electronic supplementary material, table S1), 16 amplified poorly (only short amplicons could be recovered; table 1) and 18 failed to amplify (table 1; figures 1*b* and 2). Efforts to amplify samples that failed were not exhaustive, so the success rates can be regarded as a minimum rate.

**Table 1.** Summary statistics: amino acid d/l Asx and concentration (as pmol amino acids) for each of the three ranks of DNA amplification (aDNA dataset).

rank	meaning	*n*	insufficient amino acids for extraction	mean d/l	s.d. d/l	mean conc.	s.d. conc.
2	long amplification	57	0	0.069	0.030	1.04E + 05	5.20E + 04
1	only short amplification possible	16	1	0.070	0.021	8.32E + 04	4.68E + 04
0	failed to amplify	18	4	0.065	0.014	6.62E + 04	5.51E + 04

All the d/l Asx of these bones were in the range 0.02–0.17, and the ratios between successes and failures did not vary significantly. Only six bones had d/l Asx greater than 0.1, the ratio above which amplification is thought to fail, according to the conventional criterion of Poinar *et al*. (1996). All six of these bones amplified successfully and five of these were ranked as amplification success 2 (‘good’; figure 2*a*).

Amino acid (collagen) concentration was surprisingly variable within a single bone, yet when amino acid content was compared with DNA amplification there was a significant difference between successful and failed samples (*p* = 0.016), and this difference becomes highly significant (*p* = 0.009) if the test compares ‘good’ and ‘failed’ amplifications. Based upon this comparison, ‘collagen’ concentration, despite within-bone variation, is a better method for discrimination between samples than d/l Asx (figure 2*b*).

Perhaps the most remarkable aspect of the d/l Asx values is the very narrow range of all 91 samples. In the original study of 26 bones, values ranged from 0.05 to 0.75 (Poinar *et al*. 1996) while, in our investigation, 91 samples had a spread from 0.02 to 0.17. In part, this may reflect the narrow range of burial environments (caves and permafrost).

### (c) d/l Asx in total bone, insoluble and soluble fraction (UoD) of archaeological and ancient bones

In order to explore the narrow range of d/l Asx values further, an analysis was conducted on different fractions from a range of human and animal bones spanning a greater range of burial environments and ages. In addition to measuring total bone d/l Asx (as above), bones were demineralized and the soluble and insoluble fractions were analysed separately. The results for total bone (Asx d/l = 0.044, *σ* = 0.013, *n* = 63) are slightly lower than in the first set of bone samples because of the 6 h hydrolysis at UoD. As predicted, the ‘collagen fraction’ has a low and consistent level of Asx racemization (0.036) (*σ* = 0.01, *n* = 63; figure 1*a*, insoluble fraction; electronic supplementary material, fig. S2*a*), whereas the values from the soluble ‘gelatine fraction’ (Asx d/l = 0.111, *σ* = 0.041, *n* = 63) are significantly higher and more variable (figure 1*a*, soluble fraction).

## 4. Discussion

### (a) Racemization—the role of the triple helix

In the bone specimens analysed here, the average level of racemization in the insoluble fraction was surprisingly low and surprisingly consistent, d/l Asx = 0.036 (*σ* = 0.01, *n*=91; figure 1*a*; electronic supplementary material, fig. S2*a*). There are 139 Asx in (bovine) Type 1 collagen. Subtracting the induced racemization that occurs during hydrolysis (0.004; Poinar *et al*. 1996 for serum albumin), the observed d/l Asx value of 0.032 could be explained if only eight (0.030) or nine (0.033) residues were fully racemized; there are eight residues (all Asp) in the telopeptide.

Our views on the racemization of collagen have been described elsewhere (Van Duin & Collins 1998; Collins *et al*. 1999; Ritz -Timme & Collins 2002), but racemization of Asx in ancient bones has not previously been investigated systematically. Collagen Asx racemization is unusual because of the constrained nature of the collagen helix. The *primary* structure of collagen, which comprises 90 to 95 per cent of bone proteins, is highly favourable to rapid racemization because of the high proportion of Asp and Asn residues N-terminal to Gly (Radkiewicz *et al*. 1996; Collins *et al*. 1999), yet racemization is constrained by the *quaternary* structure of the helix (Van Duin & Collins 1998). Collins *et al*. (1999) argued that racemization would be focused in the (non-helical) telopeptide region. The only telopeptide aspartic acid residue investigated in detail, αI Asp^1211^ (C-telopeptide), achieves near d/l equilibrium after 30 days of synthesis (Gineyts *et al*. 2000).

### (b) Modelling

We modelled this process (electronic supplementary material, fig. S3) using three pools of Asx residues with different rates of racemization: rapid racemization in the telopeptides (5.7%), no racemization in the triple helix (91.3%) and variable racemization of the non-collagenous proteins (3%). In our models, collagen (triple helix and telopeptides) is lost from the bone in the sigmoidal pattern observed in experimental studies (Rudakova & Zaikov 1987; Okada *et al*. 1992). The observed data broadly fit this three pool mixing model, which is sensitive to the extent of racemization in the triple helix (electronic supplementary material, fig. S3*c*), but not to the retention of the small pool of original NCPs (electronic supplementary material, fig. S3*d*) as they contribute only a small fraction of the total Asx residues.

### (c) Total d/l Asx in bone samples

Estimates of total Asx in ancient bones based upon mixing models of the extent of racemization in insoluble and soluble fractions (figure 1*a*) suggest that the contribution of the soluble fraction, although variable, was significant (11.77%, *σ* = 8.26%, *n* = 22) and much higher than the contribution from NCPs in modern bone (2.82%, *σ* = 1.53%, *n* =12; Ritz *et al*. 1994). Amino acid profiles also suggest that this fraction is predominately soluble collagen (gelatin). The low d/l Asx in all bones could be explained if the proportion of soluble collagen was always small relative to the insoluble pool, and consequently the overall d/l Asx values remain low (figure 1*a*).

Published studies of d/l Asx from bone fall into two categories: (i) samples from temperate regions, which generally show relatively high protein contents and low Asx d/l (e.g. Stone & Stoneking 1999; Caramelli *et al*. 2003; Cappellini *et al*. 2004), and (ii) samples from warmer climes, which have low protein contents and high d/l Asx (e.g. Kumar *et al*. 2000; Reed *et al*. 2003). When d/l Asx is plotted against either sequence length in bp (figure1*c*; data from Poinar *et al*. 1996) or nitrogen content, as a proxy for protein content (figure 1*d*, from a range of published data as detailed in the legend), the same ‘L-shaped’ plot is observed; d/l Asx values remain low when significant levels of amino acids are present. The high d/l Asx observed by Poinar *et al*. (1996; figure 1*c*) seem atypical. We speculate (based upon the unusual [Gly]/[Asx] values in all but four samples) that high AAR values are from bones with little residual collagen.

Our data suggest that the observed d/l Asx values of bone reflect two processes: (i) the rate of deterioration of the triple helix, which is linked to the rate of diagenesis of the mineral phase (collagen is stabilized by bone mineral; Kronick & Cooke 1996; Covington *et al*. 2008) as well as thermal age; and (ii) the rate of loss of soluble collagen, which depends upon the size and density of the bone fragment and the extent to which the burial environment promotes leaching of soluble gelatin from the bone. The kinetics of DNA depurination (Poinar *et al*. 1996) are similar to racemization of free aspartic acid (Asp) in solution, but not to the observed kinetics of Asx racemization *in bone*, which is closely related to the activation energy of collagen deterioration (173 kJ mol^−1^; Buckley *et al*. 2008).

### (d) Racemization as a tool to screen for aDNA success?

We found no correlation between d/l ratios and amplification success (figure 1*b*). If AAR is not a useful marker for DNA preservation, is there any other measure or combination of measures for amino acid preservation that predicts DNA survival?

Serre *et al*. (2004) used a combination of high ppm values (above 30 000) and low d/l Asx (below 0.1) to predict DNA survival in Neanderthal bones; we did not find any combination of values for which d/l reliably predicted DNA preservation for single bones. Despite good analytical precision for both d/l Asx and concentration (99% correlation; electronic supplementary material, figs S1*a*,*b* and S2*a*,*b*), like Reed *et al*. (2003) we found large intra-sample variability from the same bone sample (electronic supplementary material, figs S1*c* and S2*c*). The discrepancy between results from different pieces of the same bone is presumably explained by heterogeneity within the sample. The very advantage of amino acid analysis, namely that it requires only a small sample of bone, confounds its utility as a non-destructive screening method because microsampling samples this heterogeneity.

## 5. Conclusion

There is neither theoretical support, nor evidence from our data, for the use of AAR as a screening technique for DNA preservation. The original comparison was between free Asp in solution and DNA depurination; despite its elegance, this comparison has little relevance to bone. Bone Asx is dominated by collagen and Asx racemization is a measure of the state of the collagen triple helix (Weiner *et al*. 1980). Other materials will display much more rapid and progressive increases in levels of Asx d/l, including historical corals (Goodfriend *et al*. 1992) and elastin (*in vivo*; Ritz-Timme *et al*. 2003). The use of this screening method on other tissues (e.g. Marota *et al*. 2002) would have to be independently validated to establish the kinetics of Asx racemization. As an aside, it is difficult to envisage circumstances under which the extent of d/l Asx in bone could be used as a geochronological tool.
